# Robustness and rich clubs in collaborative learning groups: a learning analytics study using network science

**DOI:** 10.1038/s41598-020-71483-z

**Published:** 2020-09-02

**Authors:** Mohammed Saqr, Jalal Nouri, Henriikka Vartiainen, Matti Tedre

**Affiliations:** 1 School of Computing, University of Eastern Finland, Joensuu, Yliopistokatu 2, 80100 Joensuu, Finland; 2 Stockholm University - Department of Computer and System Sciences (DSV), Borgarfjordsgatan 12, Kista, PO Box 7003, 164 07 Stockholm, Sweden; 3 University of Eastern Finland, School of Applied Educational Science and Teacher Education, Joensuu, Yliopistokatu 2, 80100 Joensuu, Finland

**Keywords:** Network topology, Human behaviour, Computer science, Information technology

## Abstract

Productive and effective collaborative learning is rarely a spontaneous phenomenon but rather the result of meeting a set of conditions, orchestrating and scaffolding productive interactions. Several studies have demonstrated that conflicts can have detrimental effects on student collaboration. Through the application of network science, and social network analysis in particular, this learning analytics study investigates the concept of group robustness; that is, the capacity of collaborative groups to remain functional despite the withdrawal or absence of group members, and its relation to group performance in the frame of collaborative learning. Data on all student and teacher interactions were collected from two phases of a course in medical education that employed an online learning environment. Visual and mathematical analysis were conducted, simulating the removal of actors and its effect on the group’s robustness and network structure. In addition, the extracted network parameters were used as features in machine learning algorithms to predict student performance. The study contributes findings that demonstrate the use of network science to shed light on essential elements of collaborative learning and demonstrates how the concept and measures of group robustness can increase understanding of the conditions of productive collaborative learning. It also contributes to understanding how certain interaction patterns can help to promote the sustainability or robustness of groups, while other interaction patterns can make the group more vulnerable to withdrawal and dysfunction. The finding also indicate that teachers can be a driving factor behind the formation of rich clubs of well-connected few and less connected many in some cases and can contribute to a more collaborative and sustainable process where every student is included.

## Introduction

Collaborative learning is currently being implemented at all levels of education^1^. Collaborative learning is typically defined as two or more students working together toward a shared goal^1,2^. In collaborative learning, students are expected to explain their thoughts and actively communicate, discuss, and negotiate their perspectives with the other students^1^. The students also need to coordinate and regulate their interactions and contributions as well as share responsibility for both the learning process and the common product of it^1^. Accordingly, a wide spectrum of skills for communicating and collaborating effectively is considered to be an important aspect of collaborative learning^2^. While several studies have confirmed the potential benefits of collaborative learning, orchestrating and sustaining productive inter-subject interaction is often difficult to achieve^3,4^.

In a well-developed peer collaboration, the students pursue and contribute to a common goal, which requires, for example, shared epistemic agency as well as the equal distribution and organization of the workload^5–7^. The absence of one or more of these elements may fuel conflicts that can cause dysfunctional group dynamics, frustration, misunderstandings, withdrawal, decreased commitment, and even the withdrawal of one or more members^3,8^. Accordingly, the nature of the interactions and the mechanisms underlying them constitutes an important part of research into collaborative learning^2^.

Several studies have investigated the emergence of conflicts and their detrimental effect on student collaboration^3,9^. Näykki et al. used in-depth face-to-face interaction analysis of a group of collaborators and found that conflicts led to the avoidance of interaction and lower engagement^3^. Curşeu et al. studied interpersonal conflicts in collaborative groups using network density and interaction patterns to determine how conflict influences group cognition, and concluded that the management of conflict requires teachers to institute and reinforce specific structures for interpersonal interaction^9^.

Similarly, studies of the role of social network analysis in studying collaborative groups have explored a wide range of topics such as computer supported collaborative learning (CSCL)^10,11^, the mapping of interactions to identify engaged or isolated students and improve learning design^12,13^, identifying gaps in collaborative learning and designing a relevant intervention^13^ and investigating collaboration as a proxy of performance in learning analytics studies^14–17^.

However, there is a marked lack of research on the applicability of network science methods for in depth analysis of the robustness of collaborative groups: that is, their capacity to remain functional despite withdrawal, a lack of activity, or the absence of group members. The aspect of robustness in general has so far been overlooked in research on collaborative learning. Given that sustained interactions are necessary for collaborative groups and to achieve the shared goals of collaboration, studying robustness becomes important for understanding how groups (1) are functioning, (2) can be expected to continue to function, (3) can function under stressful situations and unexpected changes in group dynamics, and (4) can tolerate the attrition of group members and changes in the dynamics of interaction. Studying group robustness can also facilitate the design of better collaborative learning environments that are more diverse, encouraging of interactions, less dependent on moderators, and consequently more robust^18–21^.

This study applies methods from network science to shed light on the nature of sustainable interactions and learner network structure in collaborative learning. More specifically, the study contributes to a better understanding of the robustness of collaborative groups and demonstrates the utility of network analysis. While the structure, function, and dynamics of collaborative learning are complex, dynamic, and self-organising by nature, the methods of network science can provide new ways to make visible, scaffold and regulate interactions and collaborative learning processes as they emerge.

This study is organized as follows; the “Background” section will review the concept of robustness, discuss the value of interactions in collaborative group and how robustness of the group is essential so that the group continues to serve its function as the backbone for co-construction of knowledge. We then discuss the different dimensions of analysis of collaborative learning. In particular, we use learning theories and collaboration analysis frameworks to argue how a students’ contribution to the cohesion and robustness of the collaborative group correlates with quality of knowledge co-construction and learning gain. We then discuss the importance of robustness for the group and for the individual learner, a brief review of the rich club phenomena is then presented, followed by the aim and research questions. In the “Methods” section we describe the context, the methods, and statistical analysis, followed by the results and the discussion.

## Background

### Networks and robustness

For the purposes of social network analysis, a network is a group of actors (nodes) connected by relationships or interactions (edges). Networks are used to capture the interactions among collaborators as well as other kinds of relationships between the actors. The wiring of the connections between collaborators can determine, for example, the dynamics of interactions, the spread of knowledge, the behaviours of actors, or the distribution of resources^22–24^. Network analysis has been used to study many different aspects of collaborative learning and collaborative groups. Typical applications include the study of the patterns of interactions, the activity of collaborators, interactivity between groups, and predicting performance using learning analytics methods^10,11^.

The study of networks, or network science, offers far more possibilities beyond the study of interactions that is currently the most common. Network science offers rich, empirical, data-driven methods that have contributed to the understanding of, for instance, biological, social, and economic systems. Its methods have led to new findings in cell biology, disease mechanisms, drug discovery, and improved web searches, as well as many other applications^25,26^. Collaborative networks in learning can be interpreted as a complex, adaptive network, where the methods of network science can reveal interesting findings on collaboration, learning, interactions, and other relationships that can be modelled in network terms. According to László Barabási, “a key discovery of network science is that the architecture of networks emerging in various domains of science, nature, and technology are similar to each other […]. Consequently, we can use a common set of mathematical tools to explore these systems”^21^. The popularity and applications of network science to investigate various aspects of learning are rapidly increasing^21^.

One potential application of network science in the domain of education is to use it for studying how a collaborative group can maintain collaboration/remain functional when facing challenge. This study applies methods from network science to explore the robustness of collaborative groups in the face of the attrition of group members for whatever reason, such as conflicts or challenges, specifically by analysing the ability of the group to continue to function even when one or more actors are removed from the network. The study explores to what extent a robust group can sustain withdrawals, yet still continue to serve its intended function. The study approaches group robustness by looking at the effects of how the withdrawal of members from the group affects the potential interactions and group connectivity.

Research on robustness typically uses simulation to try and predict the ability of a system to carry out its intended functions and withstand challenges such as the loss of group members. The loss of group members or interactions in simulations can be one of two types: targeted or random^18–21^. For an example of targeted removal, think of the effects of removing a central, active study group member (involved in many interactions), or removing a moderating teacher or a leader student. Due to the key role that those members play in networks, their withdrawal or sudden inactivity can be disastrous to the network; that phenomenon is known as vulnerability due to connectivity^21^.

**Figure 1:**
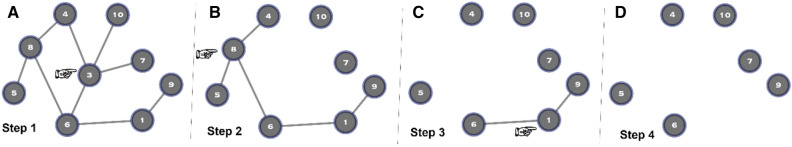
A simulation of a targeted attack, where the removal of the most connected nodes renders the network completely disconnected in 3 steps.

To illustrate the concept of how targeted removal affects a network, Fig. 1 illustrates a network of nine actors (nodes) who can be, for instance, scientists collaborating on a research project. The scientists are all connected to each other either directly (within same organisation) or to one another, through collaboration networks. The removal of scientist 3 (the most connected and active researcher based on interactions) renders two others disconnected, the subsequent removal of scientist 8 renders three more scientists disconnected, after which removal of scientist 1 renders the network completely disconnected. The network in Fig. 1 has low robustness to the removal of actors (nodes).

**Figure 2:**
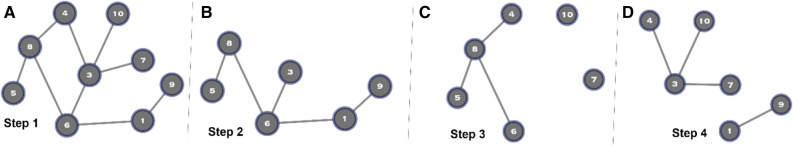
A simulation of random removal of nodes, showing the removal of random nodes.

However, the random removal of nodes does not typically lead to similarly dramatic results, and would, in most cases, take more steps to render the group of scientists in Fig. 1 disconnected. For example, removing scientists 10, 5, or 4 would have left all of the remaining scientists still connected. Figure 2 shows examples of three random node removals from the network in Fig. 1. The removal of scientists 4, 7, and 10 kept the network connected; the removal of scientists 1, 3, and 9 in Fig. 2B disconnected two scientists; and the removal of nodes 5, 6, and 8 divided the network into two components. As demonstrated in the previous example, the removal of key nodes can have a large impact on networks, as they are often hubs (greatly exceed the average number of edges) or articulation points (their removal cuts the network into two or more disconnected components).

### Robustness, collaboration and students’ learning

There is a wide-ranging acceptance that student’s reciprocal interactions, interpersonal relationships and the roles that students’ play (e.g., leaders, moderators, isolates) facilitate productive knowledge co-construction. Several learning theories support this belief such as social cognitive theory^27^, the socio-cultural framework^28^, group cognitive models^29^, community of inquiry^30^, social capital theory^31^, connectivism learning theory^32^, and socially shared regulation framework^33^. In fact, a large volume of empirical research confirms the value of collaborative learning and interactive pedagogies; a recent meta-analysis of 425 studies analysed the role of collaboration in collaborative learning and estimated a combined effect size of 0.42 on knowledge gain and an effect size of 0.64 on skill acquisition^34^, similar results have been reported by other meta-analyses and large scale systemic reviews, e.g.,^35–37^.

Researchers contend that collaborative learning has a participatory, a social and an epistemic dimension^38,39^. The participatory dimension reflects the quantity of contributions as students’ externalize their ideas, respond to each other and integrate ideas by building on to contributions of others. The participatory dimension provides valuable information about students’ engagement (both static and continuous) and can be accurately captured by SNA degree centralities reflecting the quantitative aspects of students’ contributions. Research has shown that students out-posts (captured by outdegree) correlates with students’ efforts, and performance^40–42^, while replies to students’ contributions (captured by the indegree) mediates the value of contributions. In other words, some ideas and contributions in collaborative knowledge construction may be connected, elaborated and synthesized more intensively than others^43^. Similarly, outdegree has shown positive correlation with performance^41,42,44^. However, degree measures are local centralities, i.e., only captures the immediate interactions and do not capture the breadth or range of influence or relationships through the whole network^45,46^.

The social and relational dimension in collaborative learning reflect the processes through which norms, processes and rules develop in the groups dynamics and interaction. A sound social dimension leads to a cohesive group where learners build strong interpersonal relationships, successfully assume collaborative roles and grow a strong sense of community and relatedness. A strong social structure acts as a backbone for successful information exchange and group learning success^32,38,39^. In other words, building strong, cohesive and sound interaction dynamics and relationships buttresses a robust social structure that serves as a catalyst for students’ cognitive gains and knowledge acquisition^39^. Members of a robust group help each other to participate in and contribute to collaborative work and knowledge building towards shared goals^9,47,48^. As Kreijns et al. postulates: “A performing group requires that the social space is sound. This is the case when the group structures manifest themselves by strong relationships, group cohesiveness, trust and respect, feelings of belonging, satisfaction, and a sense of community”. See Kreijns et al. for a detailed review^39^. Since the soundness, cohesion and strength of the social structure are essential elements of collaborative learning, identification of social dimension is critical for supporting productive group work.

The social dimension of collaborative learning can be captured with Eigenvector centrality and related measures, i.e., hub and authority centralities (described in the “Methods” section). These measures not only reflect the number of connections (as degree centralities) but also their strength of influence of the connections as well. Another way quantify the role of a learner in a network is to measure the drop in interactions—by simulation-that results from deletion of the learner’s interactions. For example, when an active contributor posts and get replied to, she/he engages others and stimulates the discussions, deleting these interactions—that the learner was involved in-would give an idea on the learner’s contributions and the contributions that she/he stimulated. In doing so, it reflects the role of the learners’ contributions in nurturing connections, contribution to network robustness and facilitating knowledge co-construction. An advantage of such method, is that it reflects not only the benefits one gets from the collaboration but also, how they facilitate the collaboration and their contribution to the network.

### Robustness and group function

As discussed in “Robustness, collaboration and students’ learning” section, a functioning collaborative group serves as the backbone of knowledge co-construction and information exchange among collaborators. Collaborative and cooperative interactions, such as communication activity (density of links), as well as the inclusivity of group members in interactions (redundancy of links), contribute to the interconnectedness of the group and thus robustness to the attrition of group members^11,49^. As such, the pattern of interconnectedness may reflect how a the network of collaborators adjust to absence of members. In a *decentralised* network (a network with diverse participation), there are enough links between all members that the group could lose any single member and remain connected. In some networks, a central core with a sparser periphery may emerge, known as core-periphery organization^50^. Rich-club phenomena is one of such core-periphery organizations where highly-connected nodes or *hubs* tend to interact among themselves more than they do with less connected nodes^51–53^. In a learning network, the rich club phenomena may indicate the dominance of a small subset of students or an *“oligarchy”* over the collaborative process, making it less collaborative^51,52^. Students outside the rich club may be isolated, consumers (only read others’ contributions), free-riders or low achievers. Vaquero et al. reported that high achieving students tend to build a rich club early in the course where low-achievers barely interacted and were consequently excluded from the information exchange process^54^. Recognition of such patterns is important for the group function as well as for the students who may need support or scaffolding. Furthermore, it is important to study the influence of such network organisations on robustness.

We argue that using social networks to assess the robustness of collaborative networks would help ensure that a collaborative group is functioning and can be expected to function under challenging situations. It can further advance our understanding of collaborative group robustness in order to design better collaborative environments^18–21^.

### Rational and aim

This article explores the potential of network science for capturing the robustness of groups in collaborative learning environments. Furthermore, it explores ways to study the role of collaborators, especially from the perspective of contributing to the function of the group as a whole, such as interdependence, accountability, and group processing. This study contributes to research on collaborative learning in four ways: firstly, by exploring the utility of network analysis as a method for the study of withdrawal tolerance of collaborative groups; secondly, by comparing and demonstrating the structural differences between more and less collaborative networks regarding robustness; thirdly, by demonstrating the dynamics and timeline of events of member attrition, as simulated by network methods; and fourthly, by investigating the utility of robustness parameters and robustness measures as proxies for predicting performance. In doing this, we present a case study of a course in two distinct phases: a non-collaborative phase and a collaborative phase.

The research questions of this study are as follows:

- What is the difference in structure and robustness between more and less collaborative networks?
- What are the dynamics of group member attrition regarding actors and interactions?
- How predictive of performance are robustness structures and measures in collaborative learning?

## Methods

### Context

The College of Medicine at Qassim University, has adopted an integrated problem-based curriculum. During the first three years, students work in small groups with a tutor in each group. In the fourth year, students learn through the traditional lecture-based curriculum in a single group. Students are offered online clinical problems as a method to encourage collaborative learning and clinical reasoning. The online clinical problems are case scenarios where a patient vignette is presented that includes history, symptoms and signs, and investigations. Students are asked to offer clinical reasoning, describe their approach to the case, and provide possible treatment options. The teacher posts one or two of these problems each week. The course analysed in this study, Internal Medicine, lasted for 4 months, and there were 20 case discussions. The course was divided to two midterms. In the first midterm, the course was run by the teacher as usual. The analysis of interactions in the first phase revealed three shortcomings (few interactions between students, a teacher-centric interaction pattern and the scant participation of students in information exchange). An intervention plan was developed that tried to address the issues found in the analysis. The plan included improving awareness, the training of collaborators, improving content and teacher training:

- Improving the participants’ awareness by showing them anonymous discussions and SNA visualisations of the first phase. Participants were asked to reflect upon and discuss the value of inclusive diverse interactions.
- Conducting a short training workshop for the students where group processing and social skills, interdependence skills, and positive group attitude were explained and practiced in a test environment. Learners also practiced group evaluation and metacognitive skills.
- Improving the content (case vignettes) through a collaborative script with defined goals. Students were encouraged to contribute, debate, and reason in their replies, and argue for their contributions. The teacher was also trained to moderate online discussions and facilitate collaborative discourse.
- Training the teacher to moderate online discussions and facilitate collaborative discourse.

The first midterm would serve as the non-collaborative phase in our study, and the second midterm would serve as the collaborative phase. Both phases were attended by the same students and the same teacher, on the same course. The performance was measured by exam grades, where the exam was composed of multiple choice questions, short essay questions, and written clinical case problems. Students were classified according to the university ranking into high or low achievers according to their grades: high-achievers with distinction grades higher than 80% and low-achievers with lower results.

### Data collection

Data were extracted from the online learning management system (Moodle) using Structured Query Language (SQL) queries. The extracted data included the time stamp, subject and author (ID and name) of the post, replies to the post, the author of each reply, the text of the post, any modifications of the post, and the modification time. A social network was constructed using the post data, by considering an edge from the source author of the post to the target of the post it was directed to in the thread. A directed network was compiled from aggregating all of the edges in the course. The initial midterm data were aggregated as a separate network, which was identified as the “teaching network”, as it was a network dominated by the teacher. The second set of midterm data was also aggregated, and identified as the “collaborative network” as it was participatory.

### Network analysis

The data from the interaction and the attributes of the collaborators were compiled and imported into the R statistical software environment. A network for each term was created (teaching network and collaborative network) using Igraph R library^55^. Two types of SNA analysis were performed: visual and mathematical analysis.

#### Visual analysis

Visual analysis of social networks gives an idea about the topology of the network and the interactions between the actors. In learning networks, it can reveal the central, active, and isolated actors in the group. Most importantly, it summarises a large number of interactions in an informative visualisation^22,24^. The visualisation of each network was performed with Gephi using the Fruchterman Feingold layout algorithm^56,57^.

#### Mathematical network analysis

A mathematical analysis of social networks gives a quantitative estimation of the network properties and individual centrality measures of its participants. Mathematical analysis was performed on two levels: the network level and the individual actor level^58^.

##### Network level

*On the network level* Because this study aimed to explore the utility of network analysis as a method for the study of withdrawal tolerance in collaborative groups, we used baseline (before node removal) and changes in the parameters (after node removal) that reflect: (i) the structural and participatory properties of the network (node and edge count, mean in-degree, and mean out-degree) to study how far the withdrawal of individual collaborators affects the structure of the network; (ii) the parameters that reflect the embeddedness of the group members and interactivity to study the impact on cohesion and embeddedness (density, clustering and reciprocity); (iii) the parameters that reflect the centralisation of the group and the diversity of participation (centralisation in-degree and out-degree) to study how diversity or the lack thereof contributes to robustness; and (iv) the parameters that reflect the efficiency of the network as a communication medium or the drop in efficacy after withdrawal (vulnerability and efficiency)^59–61^. (v) The parameters that reflect the signature of the rich club phenomena.

These parameters are explained in detail:

(i) *Graph level measures* represent the overall measures of actors and interactions between them^62,63^.
  *Node count* the count of actors (nodes) in each network.
  *Edge count* the count of interactions (edges) between actors in the network.
  *Mean out-degree* average number of interactions per group member (out-edges); the higher the average out-degree, the more interactions the group member has with others.
  *Mean in-degree* the average number of incoming interactions per group member (in-edges). It is calculated as the total number of interactions by the entire group compared to the total number of members of the group. Similar to the indegree, it is expected to be higher in groups with high interactivity.
(ii) *Embedding* gives a view of the overall social structure and how nodes are linked together, and embedded in their group, as well as the overall interactivity. Embeddedness can be used be a proxy for robustness: the higher the values, the more robust the network^45,46,63,64^.
  *Network density* refers to the proportion of actual interactions to the potentially possible maximum ratio among all participants (when every member has interacted with all others). Network density is useful as a measure of group interactivity and distribution of interactions among a diverse group of collaborators.
  *Clustering* or transitivity is the tendency of two actors who have a common connection; a structural network property that manifest as triangles of nodes. Network clustering is a sign of robustness as the number of alternative links grow with the number of such triangles^45,46,63,64^.
  *Reciprocity* measures the ratio of reciprocated edges, when two individuals exchange replies. Reciprocity is higher in decentralised groups, where members value the contributions of each other and exchange replies^45,46,63–65^.
(iii) *Centralisation* measures the distribution of centrality measures within the group, and is usually calculated as variation of the centrality measures of each node compared to the maximum possible. Centralisation measures are of particular importance in a collaborative context, since they measure the diversity of the centrality measures between group members. A network with high centralisation has few important nodes, such as very actively communicating students. The following measures of centralisation were considered^66^:
  *In-degree centralisation* reflects the degree to which different actors *receive* interactions. A network with high in-degree centralisation is dominated by one or some participants who receive most interactions.
  *Out-degree centralisation* measures the diversity of *outgoing* interactions. A network with high out-degree centralisation is dominated by one or a number of participants who initiate the most interactions. Highly centralised networks may be vulnerable to the withdrawal of central members.
(iv) *Efficiency* in addition to the previous measures, the measures chosen below reflect the ability of the group to function as a medium of information exchange, and resist stress or the removal of nodes:
  *Efficiency* is calculated as the inverse average of the reciprocal distances of all node paths. It offers a good estimate of the information flow through a network. Efficiency can be a useful measure of robustness and the resistance of a network to limited failure, or the removal of a limited number of actors^67,68^.
  *Vulnerability* is calculated as the relative decrease of efficiency when the actor is removed. As such, it is a measure of graph vulnerability to the disruption of information flow^20,46,59,60,69^.
(v) Rich club organization.

*Rich club coefficient (Ø)* The extent to which well-connected nodes connect to each other than what would be expected by chance. It is computed as the fraction of existing number of connections to the maximum possible among the subset of highly connected nodes (rich nodes)^51^. Since higher degree nodes in random networks may also be connected to each other by chance, the normalized rich-club coefficient (Ø_norm_) was proposed as the relative value of the Rich club coefficient compared to the average value across a null model of a group of random networks of the same size and same node degrees as in the examined networks (1,000 in our case)^53,70^. Values over 1 indicates the presence of the rich club phenomena in the studied network^53^. The boundary and size were calculated based on the method of decreasing rank ordering of nodes^71^. For each network we report the size of the rich club as the ratio of the size of the rich club core to the network size as well as the node degree corresponding to the degree of node at the periphery of the core.

*Perturbation* In addition to the above-mentioned parameters, the simulation of perturbation (actor removal) was also performed for both networks and network structural properties were compared. Perturbation was performed to study the impact of the simulated removal of members.

- *Single perturbations* This simulation looks at the effect of the removal of a single actor from a network. To test the effect of single perturbation, we removed the teacher to test the effects of removing the teacher interactions on the network, or how structural changes in the network affect other interactions and centrality measures^21,60^.
- *Grouped perturbations* In this simulation, a group of actors is removed and the properties of the graph is recalculated to analyse the effect. Removing five actors is a common method for testing the robustness of a network. The method simulates the influence of removal of an important group of significant collaborators. To investigate this effect, we removed the five most connected actors in each network and compared the structural properties of both networks before and after the grouped removal^21,60,72,73^.
- *Sequential perturbation* In this simulation, we removed nodes one by one, in the order of the most connected nodes, to analyse the effects of this removal on the connected cluster size and connectivity. Two types of sequential removals were performed: the first was based on the highest degree of connectivity, and the second was based on the highest betweenness centrality. To be able to compare the results, we also performed random node removals. Since random node removal can result in different results, we repeated the random removal simulation 1,000 times and averaged the results^21,60,72,73^.

##### Student level

Centralities important to an educational context^10,13^ were calculated for each student (actor), including measures that reflect participatory efforts and quantity of interactions (in-degree and outdegree), measures that reflect involvement in information exchange and mediation (betweenness and closeness centralities), measures that reflect the position and importance of connections (authority and hub values), and Laplacian centrality, which reflects the importance of a student role in robustness. All parameters were calculated for teaching and collaborative networks and all actors before and after perturbation and compared to assess the impact of the simulated removal of actors on collaborators. The calculated parameters are as follows^46,61,62,74–76^.

*In-strength* The number of incoming posts or replies or student’s in-degree multiplied by the weight of edges (number of duplicated edges in this study)^46,69,74,77^.

*Out-strength* The number of outgoing posts by a student, or the student’s out-degree in multiplied by the weight of edges^46,69,74,77^.

*Closeness centrality* While the sum of distances (lengths of paths) from the student to every other student in the network is called farness, the reciprocal of farness is closeness centrality. This is a measure of reachability, involvement, and inclusion in interactions with other students. There are two modes: in-closeness, which considers the incoming posts, and out-closeness, which considers the outgoing links^46,69,74,77^.

*Betweenness centrality* measures how many instances there are where the student is a bridge between two other unconnected nodes (lied *between* nodes). Betweenness centrality can be interpreted in a number of ways in learning, such as a proxy for brokerage, mediation, and helping to bring others in^46,69,74,77^, but more research is needed to make it visible and establish empirical facts on its role in collaborative learning.

*Page rank* is a variant of eigenvector centrality that has been used across a wide variety of disciplines and proved to be sensitive to structural and robustness testing. The centrality measure considers the quantity of node connections and their quality. A node may have a high page rank, not only if it has a wealth of connections, but also if the connections themselves are highly connected^46,69^.

*Laplacian centrality* quantifies the drop of sum of squared eigenvalues when an actor is removed. It carries more relational information about the actor local connectivity and density compared to other centrality measures mentioned here, and is considered a good and sensitive measure of node removal^61,76,78^.

*Authority* in contrast to in-degree, which counts the incoming interactions, *authority score* is an indication of highly connected nodes pointing to the actor. In other words, it values the number as well as the value of the incoming connections. A high *hub score* is an indication that the node has links to other high authority nodes^45^.

*Target entropy* is a centrality measure that considers the importance of a network based on the flow of information though the node; a node is more important when a message passes through a node. As such, the diversity of interactions with multiple actors increases the target entropy value^76,78^.

*Clustering coefficient* is an indication of the density of the ego network of an actor, and the degree of clustering and connection of his connected friends together. The more friends are connected to each other, the higher the clustering coefficient^20,65^.

### Statistical analysis

The data were analysed using R statistical software environment and R! Programming language version 3.52^79^. Igraph, BrainGraph and Centriserv libraries were used to calculate network structural properties and centrality values^55,80,81^. As data violated the normality assumption, Spearman’s correlation test was performed to calculate the correlation between variables. Significance levels were calculated by comparing the resulting values to a null model of 1,000 randomly generated networks matched for size, number of interactions and degree distribution^70^.

Performance prediction and feature ranking was performed using Orange toolbox. The classification was performed using five common algorithms: Random Forest, Decision Tree, Naive Bayes, Neural Network and logistic regression with tenfold cross-validation to classify students according to achievement levels (high versus low-achievers). Features were standardised (mean subtracted and divided by the standard deviation). Five ranking methods were applied to select the most relevant feature to the variable (high vs low achievers): information gain (reduction of entropy), Gini coefficient (the inequality of variables of a frequency distribution), Chi square, Anova and relief (the ability of a variable to distinguish between the tested classes)^82^.

### Ethics

The study followed the institutional and national guidelines, and was approved from the institutional College of Medicine Research centre and national regulating bodies (Qassim Regional research ethics committee). The study protocol, consent documents and the consent procedure were approved by the College of Medicine research centre and the Ethics regional committee which issued an approval of the study and that the study follows an online privacy policy that details possible use of data for research and guarantees user protection. The online privacy policy was signed by all participants and reviewed by the ethical committee. All personal data in this study were anonymised, and personal information was removed. It is also important to mention that all students were enrolled in the course and were able to complete it regardless of signing the agreement and were able to opt out of participation in this research. The researchers did not participate in teaching, grading or organising any of the courses in this study.

## Results

RQ1: What is the difference in structure and robustness between more and less collaborative networks?

There were 34 students in the course and one teacher. Thirty-four students participated in the first midterm and 33 participated in the second. The course generated 972 posts: 708 posts in the first midterm and 264 in the second. The mean degree in the teaching network was 5.20, while it was 10.76 in the collaborative network. The mean indegree was 2.60 in the teaching network compared to 5.38 in the collaborative network. While the teaching network showed more posts, they were mostly directed to the teacher. That is, all degree measures were much higher in the collaborative network.

The attributes of each network showed that the collaborative network was more interactive (the total edges (unique connections) was 183 vs. 91). The collaborative network had higher density of interactions between participants (0.16 vs. 0.08). Degree centralisation was higher in the teaching network, especially in the indegree network, where it was 0.95 with the teacher as the highest centrality value, indicating a teacher-dominated environment. Only 2% of the interactions in the teaching network were reciprocal, in contrast to 15% in the collaborative network. Furthermore, the efficiency was higher in the collaborative network (0.64 vs. 0.58). Vulnerability (a measure of drop of efficiency on removal of the important actors) was far higher in the teaching network, reaching 0.35 compared to 0.02 in the collaborative network. In brief, the teaching network showed fewer interactions between participants, was more centralised around the teacher, and was less efficient as a communication exchange medium and more vulnerable to the absence of active collaborators. Detailed statistics are listed in Table 1.

**Table 1:** Comparison of the properties of teaching and collaborative networks.

Variables	Baseline		Removed teacher		Removed 5 members	
	T	C	T	C	T	C
Actor count	35	34	30	33	16	29
Edge count	91	183	56	167	17	107
Network density	0.08	0.16	0.05	0.16	0.07	0.13
Mean in-degree/outdegree	2.60 ± 5.60	5.38 ± 3.96	1.65 ± 1.2	5.06 ± 3.54	1.06 + 0.68	3.69 + 2.21
Centralization in-degree	0.95	0.33	0.07	0.35	0.07	0.16
Centralization out-degree	0.28	0.08	0.29	0.09	0.49	0.12
Efficiency	0.58	0.64	0.38	0.63	0.46	0.59
Transitivity	0.21	0.36	0.14	0.34	0.12	0.31
Reciprocity	0.02	0.15	0.00	0.17	0.00	0.15
Vulnerability	0.35	0.03	0.14	0.04	0.52	0.02

*T* teaching network, *C* collaborative network.

### Rich club phenomena

The teaching network exhibited the rich club phenomena, where a group of well-connected students had more connections to each other, than their connections to less connected students. The rich-club coefficient (Ø = 0.24, Ø_norm_ = 1.19) was statistically significant and higher compared to a the average of 0.2 of the random networks (Null model). These values are indicative of a few select group (*n* = 10) dominating the teaching network, while they maintain the interactivity among themselves, they collaborate the least with the less interactive students. The collaborative network showed a significant and slightly lower normalized rich club coefficient (0.93), indicating a good mixing between users of different connectivity levels, see Table 2 for full details.

**Table 2:** Comparison both networks regarding rich club metrics.

Variables	Baseline		Removed teacher	
	T.	C.	T.	C.
Ø (Ø_norm_)	0.24 (1.19)*	0.33 (0.93)*	0.25 (0.97)	0.32 (0.94)*
Core size	0.29	0.76	0.26	0.67
Rank	10	26	9	22
Degree at the periphery	5	8	4	8

*T.* teaching network, *C.* collaborative network.

*Statically significant, Ø rich club coefficient, Ø_norm_ normalized rich club coefficient.

**Figure 3:**
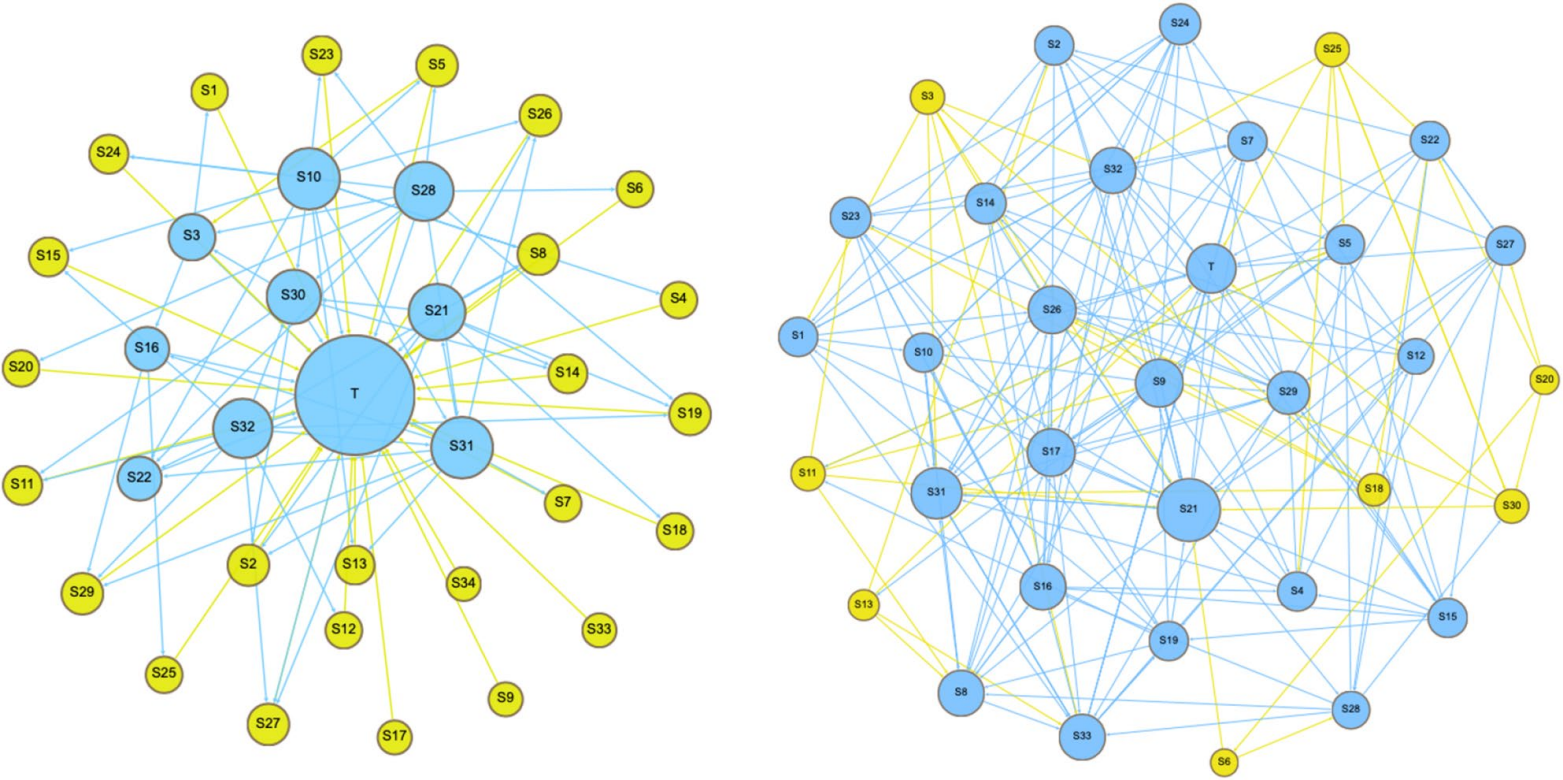
A visual plot of both networks: the teaching network on the left side with a centralised teacher as well as a rich club of collaborators (in blue). The collaborative network on the right side showing a more collaborative diverse participatory pattern, the rich club (in blue) includes the majority of students. Each circle represents a member of the group, blue colour points to members of the rich club, arrows represent direction of interactions, the node size is proportional to the degree centrality.

Figure 3 is a visual plot of both networks, the plot shows that the teaching network has a rich club of strongly connected 10 nodes (in blue) mostly centralised around the teacher, and few interactions could be seen between the rich club and the peripheral students (in yellow). In the collaborative network, the interactions are distributed among students, and there are several interconnections between students. The diversity and multiplicity of interconnections among students point to a wider diverse participation, a sign of healthy inter-subjective interactions that encouraged participation. The rich club in the collaborative network includes the majority of students.

RQ2: What are the dynamics of group member attrition regarding actors and interactions?

A common way to study the sustainability of a social system is to measure the impact of actor withdrawal, or sequentially removing important actors (perturbation). This was studied through three steps: firstly by investigating the effect of teacher absence; secondly by removing the five most active actors (in terms of degree) in each network to see how much it impacts each network’s structure and properties; and thirdly, by sequentially removing the actors from the most connected to the least connected until all edges are disconnected.

### Impact of teacher absence

**Figure 4:**
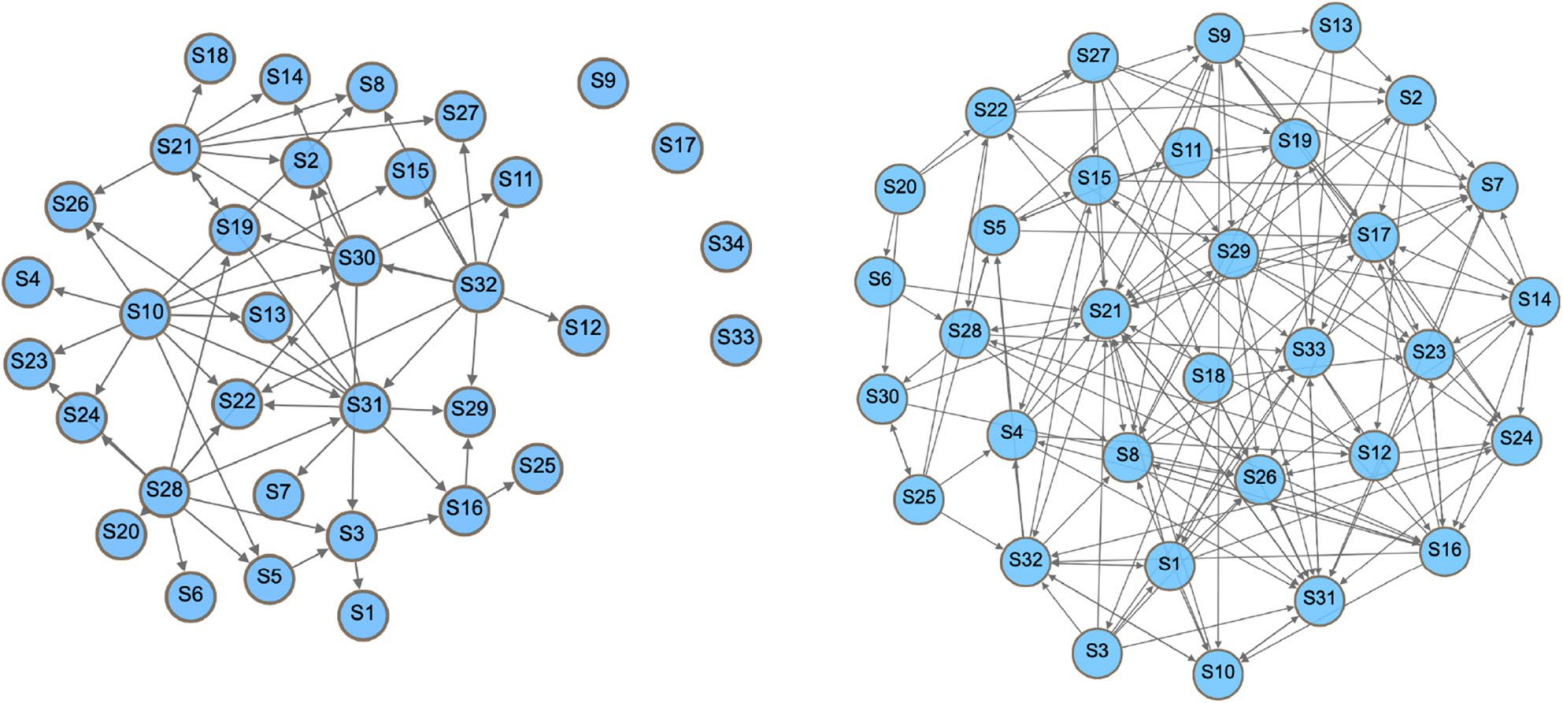
Removal of the teacher disconnected four students from the teaching network (left), while no students were disconnected in the collaborative network (right). Note the drop of interactivity in the teaching network.

Unsurprisingly, the removal of the teacher in the teaching network resulted in a marked drop in interactions (− 38%), compared to around (− 6.5%) in the collaborative network. It also resulted in four actors getting disconnected, while none were disconnected in the collaborative network. Obviously, the four disconnected students were interacting with the teacher only. Furthermore, the degree measures of interactivity fell markedly in the teaching network. Figure 4 shows a plot of both networks, and reveals a marked loss of interaction on the teaching network compared to the collaborative network after removal of the teacher (further details are presented in Table 1).

Regarding the rich club property, interestingly, the simulation of removal of the teacher has rendered the teaching network not significantly different from what is expected at random. The normalized rich club coefficient was 0.97; such change may indicate that the rich club phenomena was driven by connections to the teacher. Removal of the teacher from the collaborative network did not result in significant differences, the network has lower and statistically significant normalized rich club coefficient of 0.94 close to the null model value.

### Impact of absence of the most active actors

**Figure 5:**
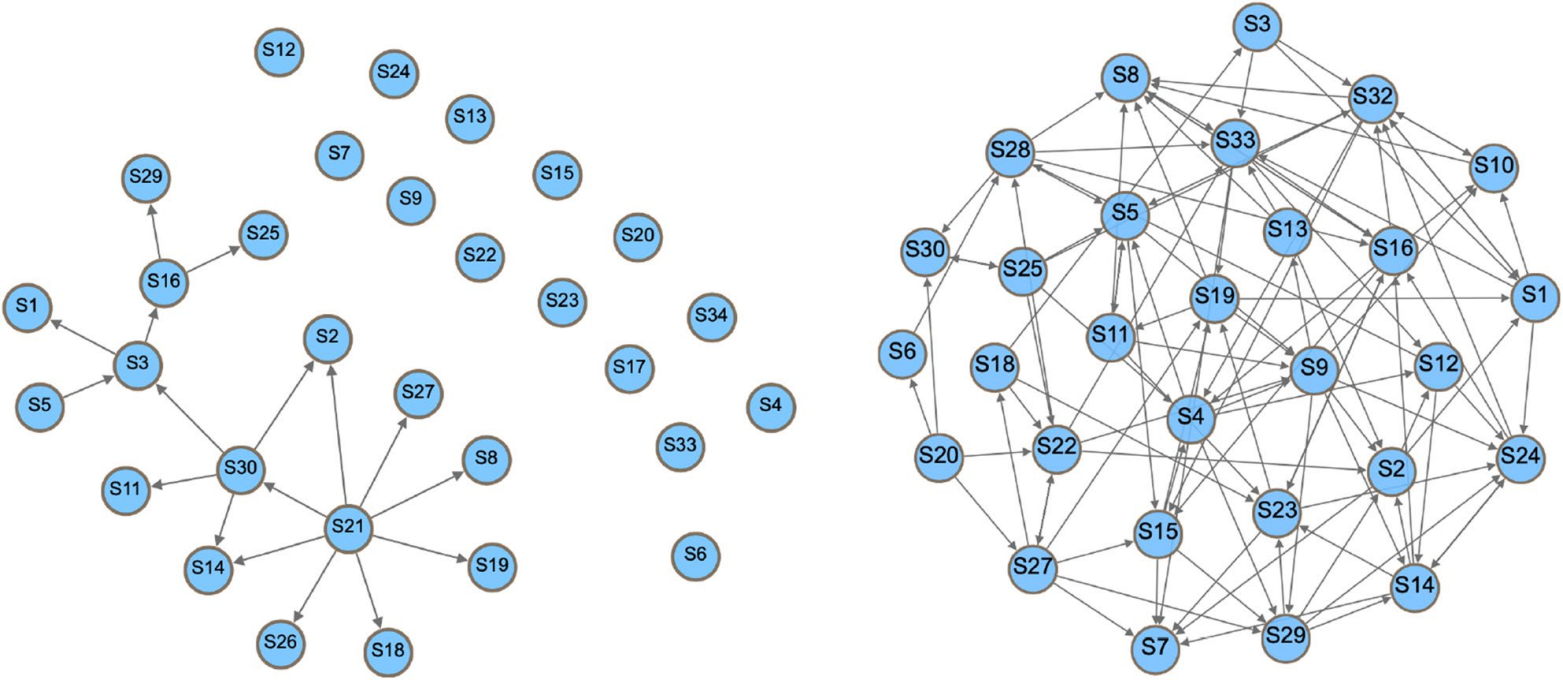
The teaching network on the left after removal of the most active students, showing 14 disconnected students and very few interactions remaining, in contrast to the collaborative network on the right which shows marked interactivity.

Removal of the 5 most connected actors resulted in disconnection of 9 others (they were only connected to these 5) and dropped the size of the teaching network by 14 actors (− 46.6%) the interactions dropped to just 17 (− 81.32%). In the collaborative network, removal of the most connected actors did not lead to the disconnection of any others, and the count of the remaining edges was 107 (− 41.53%). The removal impacted the teaching network more in terms of all degree measures, which dropped by 59% compared to 31% in the collaborative network. Centralisation measures (which measure how centralised the networks are) increased after perturbation in the teaching network, except for in-degree, which decreased in both but more markedly in the teaching network due to the previous very high index that was reliant on the teacher who was removed. The transitivity and reciprocity measures were also markedly decreased in the teaching network compared to the collaborative network. Interestingly, reciprocity dropped by 2% in the collaborative network, while it dropped by 100% in the teaching network. The efficiency of the teaching network decreased while its vulnerability increased, compared to a mild decrease in efficiency and decrease in vulnerability in the collaborative network, reflecting higher robustness of the collaborative network versus the dramatic impact on the function and interaction in the teaching network. In brief, the removal of five of the most active actors affected the collaborative network much less than it affected the teaching network; in fact, the removal of the most active actors resulted in 0% loss of other connected actors, and had only a mild impact on the interactions, keeping the network functioning and interactive. Figure 5 shows the networks after removal of the most active actors, and Table 1 shows the full statistics.

### Sequential removal

Sequential removal of most connected users was performed based on two centrality measures—degree centrality and betweenness centrality. The teaching network was completely disconnected after the removal of 9 actors based on degree of connectivity, in contrast to 27 in the collaborative network. The removal of most connected users based on betweenness centrality measures resulted in similar results: an early disconnection in the teaching network, while the collaborative network remained active until 80% of actors were removed. The two networks were similarly robust to random removal of users, indicating that networks with rich clubs may be potentially vulnerable to removal of hubs while robust against random absence of members.

**Figure 6:**
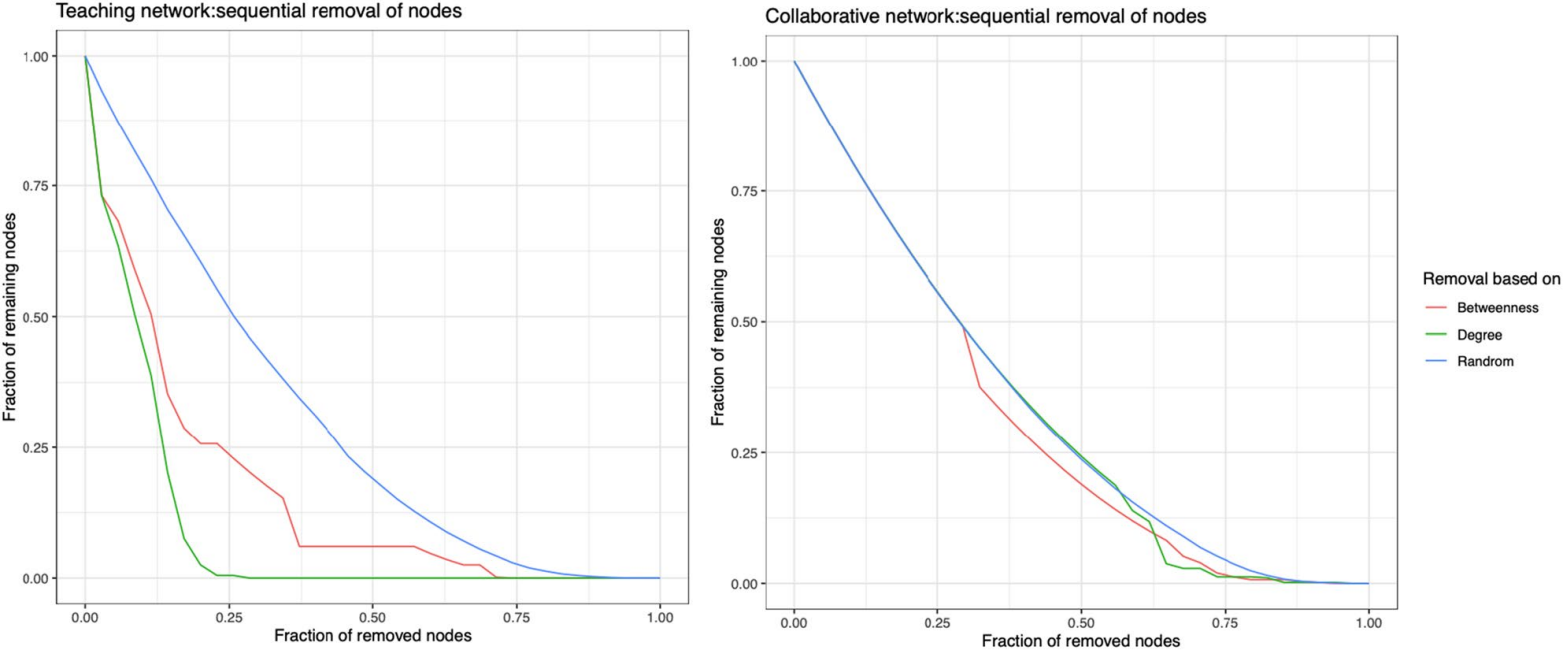
Shows the effect of sequential removal of active actors in both networks based on the highest degree (green) and highest betweenness centrality (red) compared to random removal (blue). On the left side, the connected network size dropped to 0 after the removal of 25% of actors, while in the collaborative network, the removal of the most connected actors was comparable to the random removal of actors. The simulation videos of the dynamics of sequential removal of the most active actors shows the interactions in each network after each removal.

Figure 6 shows the effect of the sequential removal of active actors in both networks; on the left side, the connected network size drops to 0 after the removal of 25% of most connected actors, while in the collaborative network, the removal of most connected actors was comparable to the random removal of actors (blue line).

Supplementary Video S1 (left) shows the a time-lapse simulation of sequential removal of most active actors, as the video shows, the removal of node 2, makes 4 others disconnected, with the exit of the next actor, other actors are consequently disconnected. The network gets completely disconnected in 9 steps. In contrast, in Supplementary Video S2 (right), the removal of the most active actor does not impact the network, and the network continues to be connected until the 27th exit of the most active actor.

RQ3: How predictive of performance are robustness structures and measures in collaborative learning environments?

To examine the relationship between social dynamics and performance, we calculated the Spearman’s correlation coefficient between grades and centrality measures. Our findings indicate that there was no statistically significant correlation among the grades and the thirteen centrality measures studied in the teaching network. In the collaborative network, we found a positive and significant correlation among grades and in-Strength (indegree with weight considered in the calculation) r(31) = 0.47, p = 0.01, in-closeness (using interactions towards the actor) r(31) = 0.43, p = 0.01, and authority score r(31) = 0.49, p < 0.01. The correlation was moderate in the four cases. As these measures are proxy indicators of incoming interactions, they highlight the value of receiving a reply from peers: a student receives an incoming connection when she/he contributes a post that stirs discussion, debate or value to be replied to. It can be interpreted as a vote by other participants who replied to these posts and emphasises quality over quantity in the teaching network. The total laplacian centrality which measures the importance in sustaining the structure of the network (or how the network communications will be impacted if the user was removed), was positively correlated with better performance r_s_(31) = 0.37, p = 0.04; this suggests that a student’s active role in promoting interactions and linking between group members is also important. In the same way, the target entropy centrality, which is a measure of the diversity of neighbours and collaborators, was also moderately statistically and positively significant r_s_(31) = 0.38, p = 0.03. The highest correlation with performance was the authority centrality, a measure of the structural importance and the strength of incoming connections r_s_(31) = 0.49, p < 0.01.

Removal of the teacher did not result in significant changes in how network indices predict performance. None of the teaching network parameters became correlated with performance and the strength of correlation in the collaborative network remained almost the same or has subtle changes. As such, connections in the collaborative network are robust to changes, and continue to predictive of performance even after removal of important actors.

See Table 3 for full statistics. In summary, although there are indications that improvements of the network structure affects the predictability of performance, the results warrant more research.

**Table 3:** Correlation among grades and centrality measures in both networks at baseline and after the removal of the teacher.

Variables	Baseline				Removed teacher			
Parameter	Teaching		Collaborative		Teaching		Collaborative	
	r_s_	*p*	r_s_	*p*	r_s_	*p*	r_s_	*p*
In-strength	0.07	0.67	**0.47**	0.01	0.09	0.64	**0.47**	< 0.01
Out-strength	0.20	0.27	0.44	0.07	0.23	0.15	0.45	0.46
In-closeness	− 0.34	0.06	**0.43**	0.01	− 0.30	0.09	**0.43**	0.04
Out-closeness	0.22	0.23	− 0.12	0.51	0.22	0.19	− 0.09	0.60
Betweenness	0.01	0.95	0.18	0.3	0.03	0.85	0.18	0.31
Authority score	− 0.17	0.37	**0.49**	< 0.01	0.28	0.88	**0.46**	0.01
Hub score	− 0.14	0.42	− 0.004	0.98	0.15	0.37	0.09	0.64
Laplacian centrality	0.21	0.24	**0.37**	0.04	0.21	0.22	**0.36**	0.04
Page rank	0.04	0.83	**0.48**	< 0.01	− 0.13	0.44	**0.48**	< 0.01
Target entropy	0.07	0.68	**0.38**	0.03	0.07	0.69	**0.38**	0.03
Transitivity	− 0.34	0.06	− 0.24	0.18	− 0.29	0.20	− 0.06	0.76

Bold values are statistically significant at the level of 0.05.

### Can robustness structures and measures be used to predict performance?

This step was performed to test whether robustness measures would facilitate the prediction of performance (classify high and low achievers) and to assess which predictors are the most significant. We compared the predictive accuracy of five common predictive models fed with the quantitative network measures as features. The features were ranked using Information gain, Gini coefficient, ANOVA, Chi square, and relief. According to the ranking, the robustness-related centrality measures (Laplacian centrality, Page rank, target entropy) were among the top predictors of performance, which confirms the results of the correlation tests—that robustness measures could be used for predicting performance. These predictors can be added to the predictors that are commonly used in learning analytics settings. In the teaching network, the top predictors were clustering coefficient, page rank, Out-Strength, closeness in, closeness out, and In-Strength. The full results of ranking are tabulated in Table 4.

**Table 4:** Comparison of feature ranking in both networks according to the ranking algorithms (Information gain, Gini, ANOVA, χ^2^ and ReliefF).

	Information gain	Gini	ANOVA	χ^2^	ReliefF
**Ranking of features in the collaborative network**					
Out-strength	0.35	0.19	0.69	2.76	0.03
Page rank	0.31	0.19	1.77	3.63	0.04
In-strength	0.19	0.12	0.98	2.38	0.03
closeness in	0.19	0.11	1.41	4.10	0.02
Laplacian centrality	0.14	0.08	0.28	2.05	0.01
Target entropy	0.09	0.05	2.15	1.75	0.05
**Ranking of features in the teaching network**					
Transitivity	0.07	0.05	0.35	0.01	0.02
Page Rank	0.06	0.04	1.53	0.01	− 0.01
Out-Strength	0.04	0.03	0.01	0.06	− 0.01
Closeness In	0.03	0.02	1.56	0.23	0.00
Closeness Out	0.03	0.02	1.72	0.42	0.03
In-Strength	0.03	0.02	0.02	0.00	− 0.01

The results of the predictive models in Table 5 clearly show that the collaborative network gained a much better predictive accuracy of students’ grades. The averaged precision ranged from 0.69 to 0.79 in comparison to 0.24 to 0.38; the AUC ranged from 0.69 to 0.76, compared to 0.17 to 0.37.

**Table 5:** Comparison of classification and predictive accuracy of predictive algorithms.

Model	AUC	CA	F1	Precision	Recall
**The collaborative network**					
Naive Bayes	0.76	0.64	0.64	0.69	0.64
Neural network	0.69	0.76	0.75	0.75	0.76
Random forest	0.72	0.76	0.75	0.75	0.76
Tree	0.74	0.79	0.78	0.79	0.79
Logistic regression	0.68	0.67	0.66	0.66	0.67
**The teaching network**					
Naive Bayes	0.29	0.37	0.37	0.37	0.37
Neural network	0.17	0.23	0.24	0.24	0.23
Random forest	0.23	0.33	0.32	0.31	0.33
Tree	0.35	0.40	0.37	0.35	0.40
Logistic regression	0.32	0.43	0.39	0.37	0.43

## Discussion

Although the issue of robustness has been extensively studied in other fields^21^ and social groups^83^, few studies have investigated the role of network methods in analysing the structure and dynamics of robustness in collaborative learning groups^11^. Understanding the structure or topology of the collaborative group is fundamental for understanding collaboration dynamics and interpersonal relationships that that underlies for students’ cognitive gains (Kreijns et al. 2013). Networks help to capture the dynamics of interactions within a complex system such as a group of people, organisations, and collaborative groups^21,49^. The study of the robustness of the networks is usually performed to improve the function of the current systems, and to design future environments that are more sustainable^21,61^. Collaborative learning networks are no exception; studying and understanding which structure is more robust against attrition and inactivity would help designers of learning environments, teachers and tutors in collaborative learning settings to orchestrate collaborative learning more effectively. Previous research on collaborative learning has shown the importance of co-regulating and coordinating collaborative actions and relationships in terms of shared goals^6,7^, however, the problem has been that the inter-subjective processes at the foundation of collaborative learning are invisible and dynamic by nature and thus very challenging for teachers to facilitate with current instrumentation.

The results of the study shed the light on how certain interaction patterns would help to promote the sustainability of the group, while other interaction patterns would make the group more vulnerable to withdrawal and inactivity. A collaborative group where all participants contribute has diversity of interactions and few hubs would sustain absence or withdrawal. Similarly, groups that have a few dominant participants or rich clubs who maintain the cohesive structure may not be sustainable. Since the teacher and the students were the same in both networks, this experiment emphasize that teachers can play different roles, a role that encourages the emergence of an interactive few and a segregated others (rich club), and another role where the teacher promotes the formation of a participatory group that is inclusive to all collaborators and therefore, robust to withdrawal or absence.

While previous studies have shown the importance of collaboration and communications skills^2,33^, the current study takes one step further by simulating the dynamics that make collaborative learning more robust when facing withdrawal or lack of activity for any reason, such as conflicts or challenging situations. In the case of the two examined groups, the analysis shows the difference of teacher-centred (rich club) network and decentralised network structures in collaborative settings. While the rich club group had more interactions and an active moderator who kept students engaged with the task, the network was centralised, and was consequently less robust. In terms of developing more robust collaborative structures, the results of the study illustrate the importance of equal team member participation, reciprocal relationships and mutual contribution highlighted in previous studies concerning collaborative learning and problem solving^2,7,33,84^. In this context, it appears crucial for the teachers to orchestrate, scaffold and encourage shared epistemic agency that fuels and sustains inter-subjective interaction and participation. As such, our findings corroborate Curşeu et al. (2012) who emphasise that the management of conflicts or withdrawal of peers requires teachers to institute and reinforce specific structures for interpersonal interaction. This study points to one such specific structure which is characterised by the robustness of the group^9^.

Another contribution of this study concerns methodological elaborations on current research on collaborative learning and problem solving. Most studies in the education context have looked at a limited number of centrality measures; the centrality measures used were mostly those that represent the activity of the learner with little relation to the role in the group. For example, degree measures count the direct interactions with peers, while ignoring the activity of the group and most importantly the diversity and importance of the collaborator in contributing to the diversity of interactions or promoting the group interactivity. Other measures such as betweenness and closeness rely on being on the shortest path or the distance to other collaborators^45^. As such, the existing commonly used centrality measures do not capture the role of collaborators in bringing diversity, group processing and functioning. The results of this study have shown that Laplacian centrality—which is a measure of importance based on the impact on group function upon actor removal—is correlated with better performance and among the top features in the performance prediction algorithm. Moreover, target entropy, a measure of diversity was correlated and predictive of performance. These findings extend the existing centralities to measures that may show a different side of interactivity, and role in group cohesion.

The present study has also visually and mathematically demonstrated the impact and dynamics of absence of important actors. Furthermore, it has shown how the seemingly active group could be vulnerable to dysfunction if an important actor was removed. The methods used in this study could be implemented in learning management systems as part of a visual dashboard. The mathematical properties could also be used to give an accurate view of the interaction in collaborating group. Properties such as efficiency, vulnerability and centralization may be of great value for teachers and educators, scaffolding the emerging interactions in collaborative group work. Moreover, the insights of this study could inform students in the co-regulation of joint efforts in a manner that encourage shared responsibility for collaborative efforts while valuing individual contributions. Consequently, the collaborative activities and interactions could be distributed in a less centralised way and create a more sustainable learning environment.

### Limitations and future directions

The main limitation of the present study is that simulation is used to study the dynamics of withdrawal. However, the use of simulation is well justified as it would ethically problematic to remove students in collaborative groups in order to investigate the research questions posed. Consequently, in real-life learning situations students may fill the gaps of the absentees or adapt to changes in the group dynamics, which this study does not capture.

For future studies, robustness can be studied in terms of how a communication breakdown between two members affects the group connectivity. An example of the former is a case of group member leaving; the latter is a case of a conflict situation between two group members or a sudden drop in one group member’s activity levels. As this paper has studied the removal of nodes (member withdrawal), another approach would be to study the withdrawal of edges (members stopping communicating with other). More studies may be needed to test the generalisability of the findings of this study in other contexts.

## Supplementary information

Supplementary Video S1.

Supplementary Video S2.
